# Movies of cellular and sub-cellular motion by digital holographic microscopy

**DOI:** 10.1186/1475-925X-5-21

**Published:** 2006-03-23

**Authors:** Christopher J Mann, Lingfeng Yu, Myung K Kim

**Affiliations:** 1 Department of Physics, University of South Florida, Tampa, FL, 33620, USA

## Abstract

**Background:**

Many biological specimens, such as living cells and their intracellular components, often exhibit very little amplitude contrast, making it difficult for conventional bright field microscopes to distinguish them from their surroundings. To overcome this problem phase contrast techniques such as Zernike, Normarsky and dark-field microscopies have been developed to improve specimen visibility without chemically or physically altering them by the process of staining. These techniques have proven to be invaluable tools for studying living cells and furthering scientific understanding of fundamental cellular processes such as mitosis. However a drawback of these techniques is that direct quantitative phase imaging is not possible. Quantitative phase imaging is important because it enables determination of either the refractive index or optical thickness variations from the measured optical path length with sub-wavelength accuracy.

Digital holography is an emergent phase contrast technique that offers an excellent approach in obtaining both qualitative and quantitative phase information from the hologram. A CCD camera is used to record a hologram onto a computer and numerical methods are subsequently applied to reconstruct the hologram to enable direct access to both phase and amplitude information. Another attractive feature of digital holography is the ability to focus on multiple focal planes from a single hologram, emulating the focusing control of a conventional microscope.

**Methods:**

A modified Mach-Zender off-axis setup in transmission is used to record and reconstruct a number of holographic amplitude and phase images of cellular and sub-cellular features.

**Results:**

Both cellular and sub-cellular features are imaged with sub-micron, diffraction-limited resolution. Movies of holographic amplitude and phase images of living microbes and cells are created from a series of holograms and reconstructed with numerically adjustable focus, so that the moving object can be accurately tracked with a reconstruction rate of 300ms for each hologram. The holographic movies show paramecium swimming among other microbes as well as displaying some of their intracellular processes. A time lapse movie is also shown for fibroblast cells in the process of migration.

**Conclusion:**

Digital holography and movies of digital holography are seen to be useful new tools for visualization of dynamic processes in biological microscopy. Phase imaging digital holography is a promising technique in terms of the lack of coherent noise and the precision with which the optical thickness of a sample can be profiled, which can lead to images with an axial resolution of a few nanometres.

## Background

Dennis Gabor invented holography in 1948 while attempting to improve upon the resolution of electron microscopy. Although at the time this new invention was a significant advance, the requirement of a coherent light source precluded its practical use. The invention of the laser and the introduction of off-axis holography provided the critical elements to make holography a practical and powerful tool for large areas of applications from metrology, data storage, optical processing, device fabrication, and even fine arts. While the use of lasers and the off-axis technique made holographic recording and reconstruction a valuable and practical technique, the conventional process of performing holography using photographic plates is time-consuming and cumbersome. Real time processing of the hologram is not feasible unless one uses photorefractives and other nonlinear optical materials.

Recently, the field of holography has been undergoing another paradigm shift by electronic image capture using CCD array cameras and digital processing of the holographic images [1,2]. With the development of higher performance CCD and computational techniques, digital holography is fast becoming an increasingly attractive alternative to conventional film-based holography. It offers a number of significant advantages, such as simple, fast image acquisition and the availability of many powerful digital processing algorithms. By calculating the complex optical field of an image volume, the amplitude and phase of the optical field are available for direct manipulation [3-7]. There are numerous digital processing techniques for manipulating the optical field information in ways that are difficult or impossible in real space processing. For example, optical system aberration can be numerically corrected[8,9] and holographic interferometry can be performed between remotely situated objects through telecommunication links[10]. We have recently introduced digital interference holography, where short coherence lengths can be synthesized for optical tomography by superposition of many holograms using different wavelengths[11].

The applications of digital holography to microscopy are particularly advantageous because in conventional microscopy the very narrow depth of focus requires mechanical scanning of the focal plane, whereas a single hologram records the information of a three-dimensional object space. These advantages have been noted since early in the development of digital holography and applied to imaging analysis of microstructures[12,13] and biological microscopy [14-18]. In contrast with other interferometric configurations and interference microscopy which require the phase to be measured through the process of multiple image acquisition and phase modulation, digital holography requires a single image (hologram) and no phase modifying devices in order to obtain phase information. This is a significant advantage for real-time living cell analysis.

In this paper we present the results of our recent experiments on digital holography for biological microscopy. Cellular and sub-cellular features are imaged with diffraction limited resolution of 0.5 μm and the optical thickness profile is determined from the analysis of the measured phase information. As examples to determine the resolution of the optical system as well as illustrate the unique properties of digital holography we examined a standard optics test object, the USAF 1951 resolution target and a diverse range of biological objects such as red blood cells, mouse embryo fibroblast cells, and paramecium. To show the adaptability of digital holography in dynamic imaging of living specimens and cells, movies of holographic amplitude and phase images were created from a series of holograms and reconstructed with numerically adjustable focus.

## Methods

**Figure 1:**
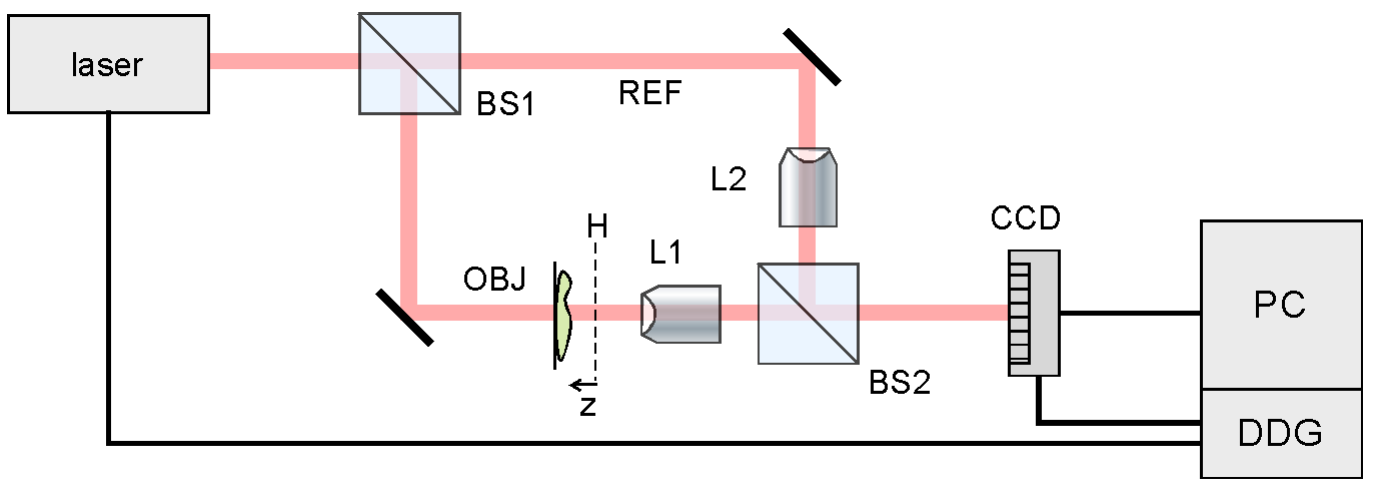
**Apparatus for digital holography experiments**. BS = Beamsplitter, L1; L2 = Microscope objectives, DDG = Digital Delay Generator, REF = Reference beam, OBJ = Object beam; H = Hologram plane;

The setup for the recording of digital holograms is depicted in Fig. 1. The 532 nm coherent light from a miniature pulsed YAG laser (Continuum Minilite) operating at about 2 mJ per Q-switched 10 ns pulse is used for hologram recording. The laser output, spatial-filtered and collimated, is split into reference and object beams in an interferometer based on the Mach-Zender configuration. A magnified image of an object specimen is projected onto the CCD camera, as well as a similarly magnified reference beam. A pair of similar microscope objectives, either (20× 0.4NA) or (40× 0.65NA) depending on the desired lateral magnification, are placed in the two optical branches to match the curvatures of the two wave-fronts. The camera (Sony DFW-V500) has an array of 640 × 480 pixels on 4.7 × 3.6 mm^2 ^active area, with 8-bit gray scale output, and the electronic shutter speed of the of the camera is usually set at 1/30 sec. A digital delay generator (Stanford Research DG535) triggers both the laser and the camera at a repetition rate of 20 Hz. An IEEE1394 cable connects the camera to the desktop computer, which processes the acquired images and calculates the holographic diffraction using a number of programs based on LabVIEW^® ^and MatLab^®^.

The hologram which is created by the interference between the object and reference fields, |*H*|^2 ^is recorded by the camera and consists of;

|*H*|^2 ^= |*O*|^2 ^+ |*R*|^2 ^+ O^*^R + OR^* ^    (1)

where *O *and *R *are the object and reference fields, respectively. Separate exposures and subtraction of the zero-order background |*O*|^2 ^and |*R*|^2 ^helps to reduce the zero order term significantly. Once |*O*|^2 ^and |*R*|^2 ^are removed the holographic twin image O^*^R + OR^* ^terms remain. A slight angle is introduced between the object and the reference beams by tilting the beam splitter BS2 in order to separate the conjugate twin images, O^*^R + OR^* ^for an off-axis hologram. The object is moved back a distance z from the focal plane of the CCD to create the out of focus image of the object.

### Numerical diffraction

Once the hologram has been captured the optically diffracted field is numerically propagated by use of reconstruction algorithms to calculate the optically diffracted field [19-22]. Here we briefly describe some of the commonly applied methods of reconstruction.

### Huygens convolution method

In the convolution process [23] the reconstructed complex wave-field *E*(*x*, *y*) is found by



and the convolution is

= *E*_0 _(*x*, *y*) ⊕ *S*_*H *_(*x*, *y*, *z*)     (3)

= **F**^-1^[**F **(*E*_0_)·**F **(*S*_*H*_)]

where *S*_*H *_is the Huygens PSF



The whole process requires three Fourier transforms, which are carried out using the FFT algorithm. The pixel sizes of the images reconstructed by the convolution approach are equal to that of the hologram. In order to achieve as high a lateral resolution as possible, one keeps the object-hologram distance as short as possible, but the discrete Fourier transform necessitates a minimum distance such that:



where *a*_*x *_is the frame size and *n*_*x *_is the number of pixels in *a*_*x*_. At too close a distance, the spatial frequency of the hologram is too low and aliasing occurs. Normally the object is placed just outside this minimum distance.

### Fresnel transform method

The Fresnel transformation is the most commonly used method in holographic reconstruction. The approximation of spherical Huygens wavelet by a parabolic surface allows the calculation of the diffraction integral using a single Fourier transform. The PSF can be simplified by the Fresnel approximation as:



and the reconstructed wave-field is:



The pixel resolution Δ*x *of the reconstructed images determined directly from the Fresnel diffraction formula will vary as a function of the reconstruction distance *z *as:



where *N *is the number of pixels and Δ*x*_0 _is the pixel width of the CCD camera. As with the Huygens convolution method there is a minimum z distance requirement set by Equation 5.

### Angular spectrum method

The angular spectrum method of reconstruction has the significant advantage that it has no minimum reconstruction distance requirement as is this case for the Fresnel and convolution methods[24]. If *E*_0_(*x*_0_, *y*_0_;0) is the wave-field at plane *z *= 0, the angular spectrum *A*(*k*_*x*_, *k*_*y*_;0) at this plane is obtained by taking the Fourier transform:

*A*(*k*_*x*_, *k*_*y*_;0) = ∬*E*_0_(*x*_0_, *y*_0_;0) exp[-*i*(*k*_*x*_*x*_0 _+ *k*_*y*_*y*_0_)]*dx*_0_*dy*_0 _    (9)

where *k*_*x *_and *k*_*y *_are corresponding spatial frequencies of *x *and *y*. Fourier-domain filtering can be applied to the spectrum to block unwanted spectral terms in the hologram and select a region of interest corresponding only to the object spectrum. Subsequently the wave-field *E*_0_(*x*_0_, *y*_0_;0) can be rewritten as the inverse Fourier transform of its angular spectrum,

*E*_0_(*x*_0_, *y*_0_;0) = ∬*A*(*k*_*x*_, *k*_*y*_;0) exp[*i*(*k*_*x*_*x*_0 _+ *k*_*y*_*y*_0_)]*dk*_*x*_*dk*_*y *_    (10)

The new angular spectrum at plane z, *A*(*k*_*x*_, *k*_*y*_;*z*) is calculated from *A*(*k*_*x*_, *k*_*y*_;0) as

*A*(*k*_*x*_, *k*_*y*_;*z*) = *A*(*k*_*x*_, *k*_*y*_;0) exp[*ik*_*z*_*z*]     (11)

where 

The reconstructed complex wave-field of any plane perpendicular to the propagating *z *axis is found by

*E*(*x*, *y*;*z*) = ∬*A*(*k*_*x*_, *k*_*y*_;*z*) exp[*i*(*k*_*x*_*x *+ *k*_*y*_*y*)]*dk*_*x*_*dk*_*y *_    (12)

Two Fourier transforms are needed for the calculation in comparison to the one needed by the Fresnel transform. However once the field is known at any one plane, only one additional Fourier transform is needed to calculate the field at different values of z. This method allows frequency-domain spectrum filtering to be applied, which for example can be used to block or remove the disturbance of the zero order and twin image components. A signficant advantage of the angular spectrum is that there is no minimum z reconstruction distance requirement.

## Results

**Figure 2:**
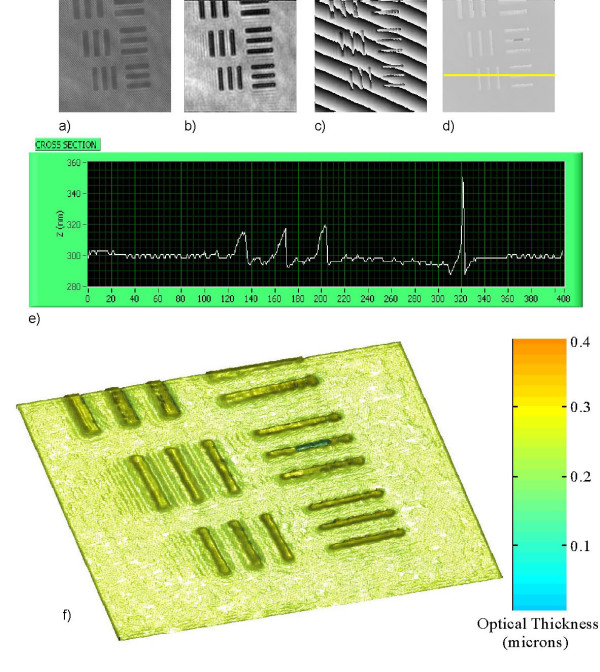
**Holography of a USAF 1951 resolution target, group 7**. The image area is 50 × 50 μm^2 ^(408 × 408 pixels) and the image is at z = 3μm from the hologram: (a) hologram; (b) amplitude; (c) wrapped phase image; (d) unwrapped phase image, (e) z-profile of optical thickness cross section and (f) 3D pseudocolor rendering of (d).

Here we present some examples of quantitative phase images obtained from digital holography experiments that examine the lateral resolution of the optical system and demonstrate the quantitative and qualitative capabilities of digital holography for microscopy. The holograms are recorded at a distance z from the focal plane of the CCD camera and thus are not recorded in focus. In each case the reconstruction distance for best focus is determined by both human observation and a contrast measurement algorithm to check for accuracy. The reconstruction here is performed by application of the angular spectrum algorithm which has no set minimum reconstruction distance. Figure 2 displays digital holographic images of a resolution target, where the smallest resolvable bars are that of group 7, element 6 with line width of 2.2μm. The observed resolution is consistent with that of the Abbe criterion for diffraction limited imaging of the optical system

Δ*ξ *= 0.61*λ*/*N*.*A *~ 0.81 *μm*.     (13)

The resolution target used is a positive mask with opaque chrome film on a transparent glass plate. The physical thickness of film is given by

*d *= *λ*(Δ*φ*/2*π*)/(*n *- *n*_0_)     (13)

where *λ *is the wavelength, Δ*φ *is the phase step, and *n*-*n*_0 _is the index difference between the film and air. From the measured optical path length change it is possible to determine either the optical thickness or refractive index provided knowledge of one or the other is known.

Quantitative phase imaging is particularly effective in digital holography – one only needs to plot the phase of the calculated complex optical field, as shown in Fig. 2c). In Fig. 2d) we demonstrate the use of a flood-fill phase unwrapping algorithm to remove the 2*π *ambiguities in the wrapped phase image and apply a linear phase offset correction to the image in Fig. 2c). The optical path length resolution was determined quantitatively by analysis of the noise level in the flat area, which corresponds to glass thickness variation of around 5 nanometers as shown in the z-profile cross section Fig. 2e) (On the film-coated bar areas, the lack of light causes larger uncertainty in phase.). Pseudocolor 3D rendering of 2d) is shown in 2f). The noise in the phase image is not dependant on the quality of the beam profile (interferometric arrangement) and is significantly less than that of the noise observed in the amplitude image Fig. 2b).

**Figure 3:**
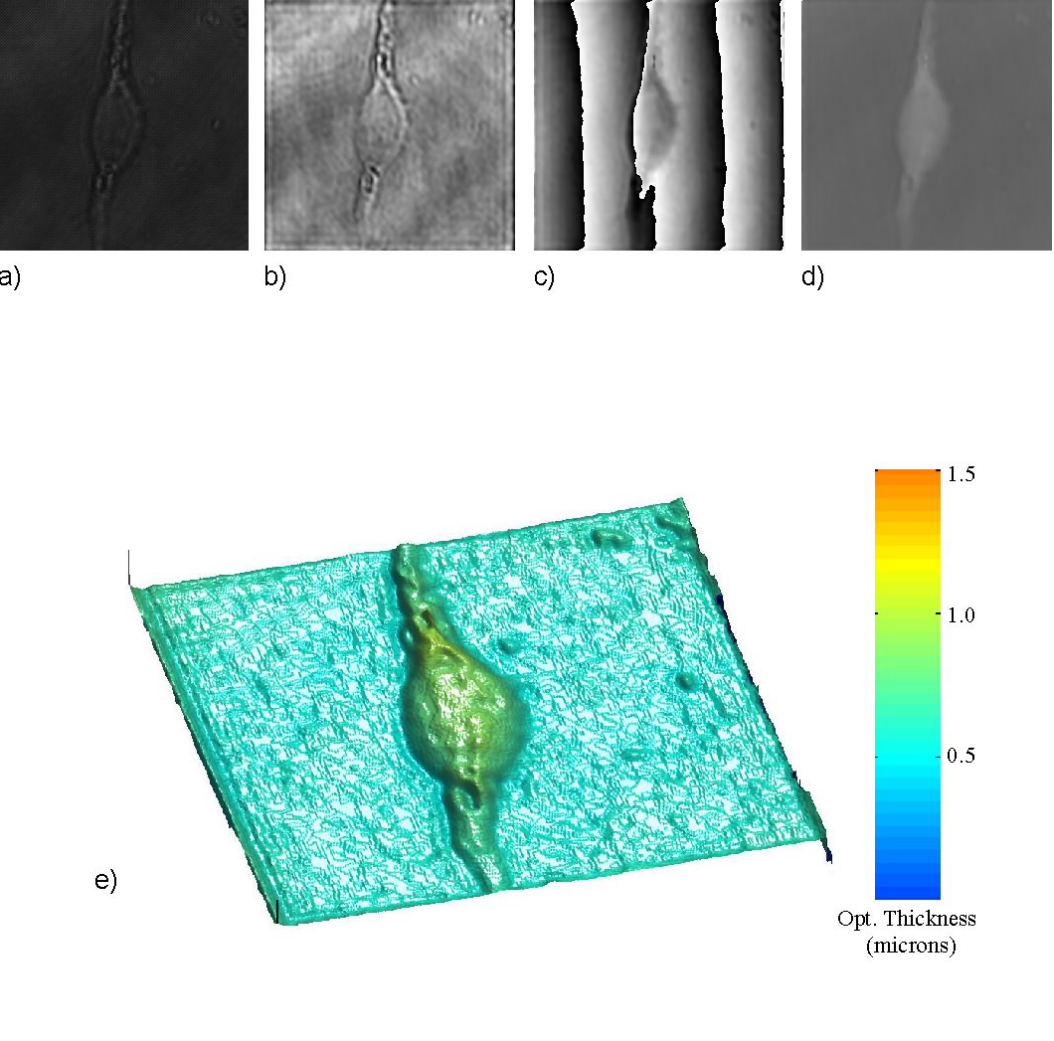
**Holography of a mouse embryo fibroblast cell**. The image area is 60 × 60 μm^2 ^(424 × 424 pixels) and the image is at z = 14μm from the hologram: (a) hologram; (b) amplitude image; (c) wrapped phase image; (d) unwrapped phase image and (e) 3D pseudocolor rendering of (d).

**Figure 4:**
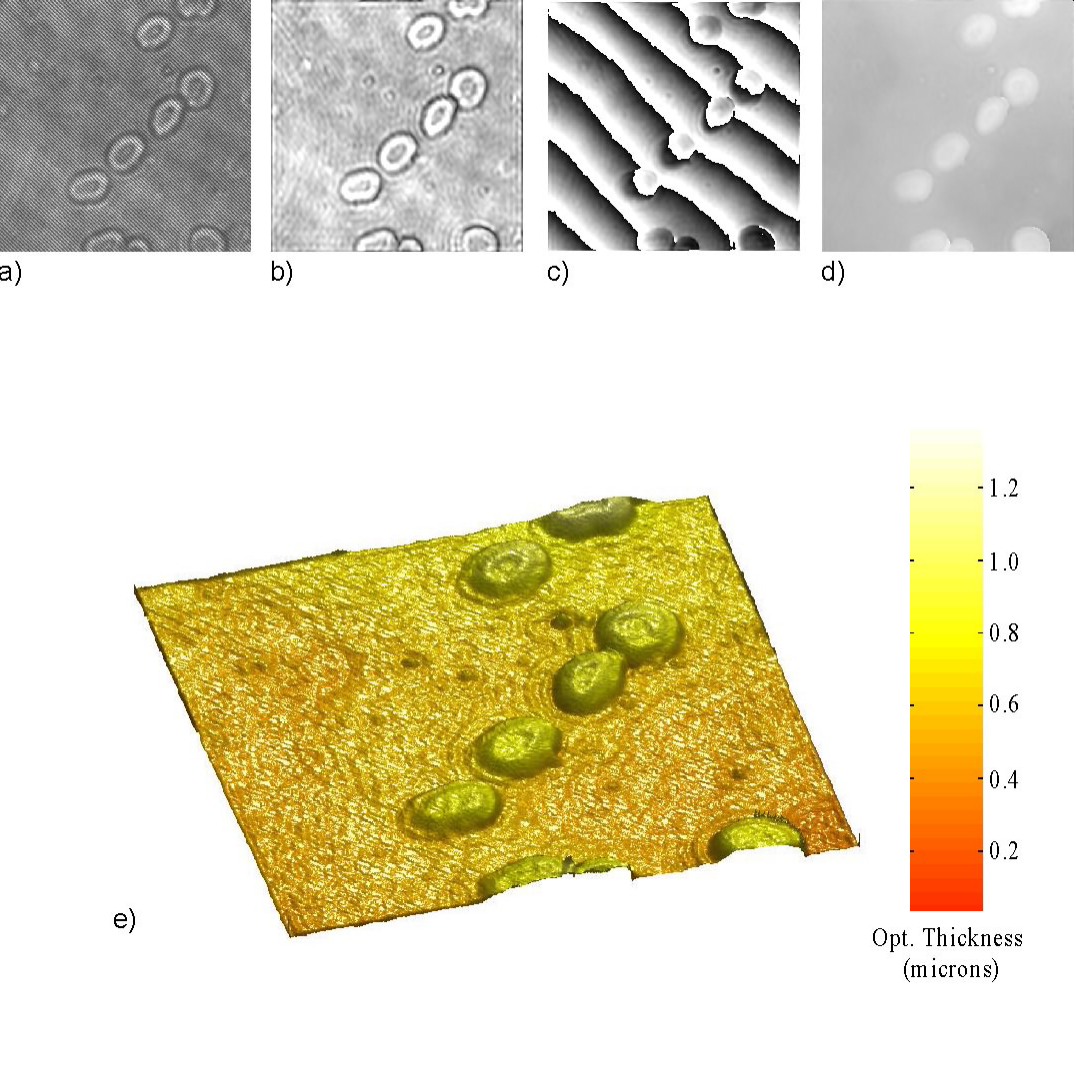
**Holography of a red blood cell**. The image area is 50 × 50 μm^2 ^(408 × 408 pixels). The image is at z = 4 μm from the hologram: (a) hologram; (b) amplitude image; (c) wrapped phase image; (d) unwrapped phase image and (e) 3D pseudocolor rendering of (d).

In Figure 3 we show images of a living mouse embryo fibroblast cell. Figure 3e) the Pseudocolor 3D rendering of 3d) shows the high quality and contrast of surface detail on the cell obtained with phase imaging and provides an accurate profile of optical thickness. In Figure 4 we show images of red blood cells. The surrounding medium is air and we apply a constant index of refraction estimate of 1.375 for the blood cell [25]. Therefore any changes that occur in the phase come from changes in the cell optical thickness *d *as seen from Equation 13. From the analysis of the phase map Fig. 4e) we infer the average optical thickness of the blood cells to be around 0.6 μm.

As well as obtaining both amplitude and phase information, another advantage of digital holography is the ability to calculate the optical field at any number of image planes from a single hologram, emulating the mechanical focusing control of a conventional microscope. To illustrate this focusing ability we show a sequence of twenty one images of a paramecium with image area 90 × 90 μm^2 ^and calculated in the range of z = 50 ~ 250 μm in steps of 10 μm. The sequence of images is then composed into an AVI movie (see Additional file 1). As the focus is scanned, one observes the food vacuoles as well as the cell wall passing through the various focuses.

### Digital holographic movies

A digital hologram contains information of the whole optical field in a three-dimensional image volume, which allows for calculation of the image field on any focal plane. A time series of digital holograms then provides complete four-dimensional information of the object's three-dimensional spatial images as well as the time evolution of those images. Once a movie of digital holograms are recorded, one can reconstruct the images of the object and be able to focus on any focal plane as a specimen under observation moves up and down in the image space. This is the most unique capability of digital holographic movie in contrast to conventional video microscopy, where only the images of the specific focal plane that was used in recording are preserved and the information of all the other planes is lost. This is a critical advantage especially in the microscopic recording of animated microbes that are constantly swimming in and out of a large range of focal distances. In conventional video microscopy, it is not feasible to track the focal distances of rapidly moving microbes, whereas in digital holography a series of holograms can be recorded at a fixed distance and the images are later numerically focused at leisure and one is able to track the rapidly moving microbes as needed. We have recorded a series of holograms by synchronizing the camera with the laser pulses under the control of the digital delay generator. The amplitude and phase images are calculated by the Angular Spectrum method while adjusting image distances for best focus on the object under observation. The reconstructed images are then composed into AVI files.

(Additional file 2) shows the hologram movie of a paramecium swimming around with a number of euglenas. The amplitude and phase images are reconstructed from the holograms while adjusting the image distances over a 20 μm range in order to maintain the paramecium in focus (see Addition files 3 and 4). The reconstruction rate from each recorded hologram is achieved in around 300 ms. Higher magnification hologram, amplitude, phase and phase unwrapped movies are shown in (Additional files 5, 6, 7, 8) each with an image area of 80 × 80 μm^2^. In order to maintain the paramecium long enough within the field of view, the paramecium is slowed down using a drop of thickening agent methylcellulose. In the amplitude movie, one observes the flickering due to celial motion in the oral groove that forces food (bacteria) into the food vacuoles. A notable phenomenon in the phase movie is the shrinking of the contractile vacuole as it pumps out water. (Additional files 9, 10, 11, 12, 13) are the hologram, amplitude, phase, phase unwrapped and 3D optical profile movies of image area 170 × 170 μm^2 ^showing selected frames of the migration process by living mouse-embryo fibroblast cells (3T3).

## Discussion and conclusion

We have presented experimental results that demonstrate the capabilities of digital holography for biological microscopy. A series of holograms are recorded and the images are reconstructed with numerically adjustable focus so that the moving objects can be accurately tracked. The holographic reconstruction is carried out by the Angular Spectrum method in an off-axis configuration so that the twin images and zero-order background can be subtracted out using frequency domain analysis. The lateral and longitudinal resolutions obtained are consistent with diffraction limited imaging. Digital holography and movies of digital holography are seen to be a useful new tool for biological microscopy, with noteworthy advantages over traditional microscopic techniques for biological imaging.

The speckle noise of a coherent imaging system is a significant issue for biological microscopy where there is often a large range of structural scales. For example, in the presented holographic amplitude images of paramecium the intracellular regions contain various assortments of unresolved particles, which may be food particles or other organelles. These degrade the quality of images to various degrees. On the other hand, one also notices that the phase images tend to suffer from the coherent noise to a significantly lesser degree compared to the amplitude images. We note that in this study we have presented the images with minimal post-processing, the goal being the demonstration of digital holography processes. Other than the overall brightness and contrast adjustments, we have not applied any of the numerous image enhancing techniques that are available[26], which can significantly improve the perceived image quality for biological applications. This will be the subject of a future study.

Phase imaging digital holography is particularly promising in terms of the lack of coherent noise and the precision with which the optical thickness can be profiled, which can lead to images of high axial resolution of a few nanometres. However the phase images are often required to be unwrapped in order to simplify their interpretation. Of particular significance is the 2π ambiguity problem in phase-imaging holography. A conventional approach is to apply one of many phase-unwrapping algorithms as we have done in this paper, but often these require both substantial user intervention and strict requirements on the level of phase noise and the phase discontinuity. They also have difficulty in correctly interpreting the phase images of rough and irregular objects. We are currently investigating multi-wavelength phase-imaging digital holography[27] for generating movies of phase-unwrapped images by using two or more laser wavelengths for illumination of the holographic system without the addition of any noise in the image.

## Supplementary Material

Additional File 1 Numerical focusing of paramecia from a single hologram. Click here for file

Additional File 2 Hologram movie of a paramecium and euglena. Click here for file

Additional File 3 Amplitude movie of a paramecium and euglena. Click here for file

Additional File 4 Phase movie of a paramecium and euglena Click here for file

Additional File 5 Higher magnification hologram movie of a paramecium Click here for file

Additional File 6 Higher magnification amplitude movie of a paramecium Click here for file

Additional File 7 Higher magnification phase wrapped movie of a paramecium Click here for file

Additional File 8 Higher magnification phase unwrapped movie of a paramecium Click here for file

Additional File 9 Hologram movie of mouse-embryo fibroblast cells in the process of migration Click here for file

Additional File 10 Amplitude movie of mouse-embryo fibroblast cells in the process of migration Click here for file

Additional File 11 Phase movie of mouse-embryo fibroblast cells in the process of migration Click here for file

Additional File 12 Phase unwrapped movie of mouse-embryo fibroblast cells in the process of migration Click here for file

Additional File 13 Three-dimensional optical thickness profile phase movie of mouse-embryo fibroblast cells in the process of migration Click here for file
